# Planning and managing for resilient natural resources and communities in the USA: the EPA Organon

**DOI:** 10.1007/s42532-025-00224-1

**Published:** 2025-08-07

**Authors:** Jordan M. West, Caitlin A. Gould, Candace K. May, Chris P. Weaver

**Affiliations:** Office of Research and Development, U.S. Environmental Protection Agency, Washington, DC United States

**Keywords:** Adaptation, Resilience, Natural resources, Human health, Management planning, Decision making

## Abstract

The EPA Organon is a collaborative framework for resilience planning created by the U.S. Environmental Protection Agency (EPA) to advance partner-centered collaborations to protect human health and the environment. An “organon” is a thought instrument, and in the case of the EPA Organon is comprised of an organizing structure, guiding principles, information resources, and project examples that are used to improve coordinated flow of information between science and implementation to inform continuous evaluation and improvement of adaptive management outcomes. It does so by providing basic orientation to key principles of resilience-based planning and action, a structure for iterative program evaluations to fill gaps and improve integration of work streams, and a logic model for working with subject matter experts on strategic design of projects on the ground. This paper introduces the Organon as a practical tool for integrated environmental and social science research, including its structure, content, flow process, and preliminary results of partner applications to date in the areas of natural resource management and inclusive collaboration. Lessons learned and next steps for expanding the Organon’s use into the spheres of public health and evaluation are also discussed, along with reflections on the value of this type of planning system for the broader adaptation community of practice.

**Supplementary Information:**

The online version contains supplementary material available at 10.1007/s42532-025-00224-1.

## Introduction

The EPA Organon is a collaborative framework for resilience planning created by the U.S. Environmental Protection Agency (EPA). The term “organon” epitomizes a structured approach to logic and reasoning that goes beyond a basic framework to also include a body of principles and resources that are applied in the context of learning and investigation.^1^ As such, the Organon is not meant to replace planning frameworks of other organizations; rather, it is an internal translational structure to rapidly achieve a common understanding among diverse partners of the status and needs of ongoing science to meet shared environmental objectives. To that end, the Organon comprises an organizing structure, guiding principles, project examples, and information resources for building adaptation and resilience into environmental and human health activities. It is being used in research collaborations to help resolve disconnects among different scales, types, and levels of science, decision-making, and partner engagement, and to overcome barriers and create openings for action.

### Context for using the Organon

The EPA’s mission is to protect human health and the environment. Fulfilling this mission requires efficient and effective methods for working collaboratively with communities to ensure their safety, well-being and prosperity in the face of environmental hazards. To advance this goal, the EPA’s Office of Research and Development promotes innovations in resilience science–and our national understanding of how communities and ecosystems can benefit–through improved integration of environmental and social sciences. This involves combining broad multi-disciplinary expertise in ecology, environmental health, public health, and atmospheric chemistry with diverse areas of social science such as anthropology, sociology, geography, and environmental studies. A core objective is to provide support to community, Tribal, state, and regional partners to boost their resilience (ability to resist and recover) when dealing with environmental changes and threats.

### Challenges addressed by the Organon

Successful resilience-based planning and implementation requires moving science into action within specific decision contexts dictated by organizational missions and stakeholder needs. Since ongoing, large-scale environmental changes present critical challenges to environmental quality and public health, agencies and the ecosystems and communities they serve must adapt to continue ensuring clean air, water, and land and associated benefits. To be effective, adaptation must be based on the best available science. That science is complex, encompassing interactions and feedback loops among human and natural systems and environmental media across a range of time scales. Consequently, the appropriate knowledge base for supporting any given instance of adaptation planning and implementation will be correspondingly interdisciplinary, cross-media, and multi-scalar.

A key challenge, however, is that the levers of environmental management for this science-based adaptation often derive from statutes and programs that, far from embodying holistic systems thinking, narrowly address individual environmental media (e.g., air *or* water), or individual environmental stressors (e.g., thermal changes *or* chemical pollution). In other words, while such problems are not stove piped, environmental management often is. Effective adaptation, therefore, depends on an intentional process of knowledge assembly, disassembly, and reassembly around a changing environmental system on the one hand, and on the other, a collection of management tools and levers available to achieve desired adaptation outcomes.

This challenge is compounded by related issues associated with scaling, generalizability, prioritization, and effectiveness. While deep place-based work carried out in collaborations among scientists and stakeholders is the surest path to effective adaptation, such work is time-consuming and labor-intensive. Experts and programs cannot be everywhere at once, so there is a need to scale up, generalize from place-based work, and transfer what works in one place to other locations that might benefit from the newfound insights. Similarly, in an era of limited resources, we must prioritize the most effective solutions, systematically evaluating successes and failures to see what works and to focus efforts for maximum leverage.

### Core attributes of the Organon

A growing body of knowledge, developed over years of scholarship and practice at the EPA, has highlighted the most critical system attributes needed to overcome the above challenges for both environmental and public health outcomes. These include built-in features that add structure and intentionality to processes, make evolving best practices iterative, and create transparency and accountability to bolster strong collaborative relationships. Achieving this requires a framework and guiding principles for integrating adaptation and resilience into environmental management, through inclusive partner-centered approaches, and in a way that can be evaluated for effectiveness.

Here, we describe how the Organon embodies these emergent attributes, illustrate its operation through examples and case studies, and describe next steps for building on progress to date. This includes expanding the scope and reach of the Organon across a widening range of environmental and public health adaptation planning, implementation, and evaluation contexts.

## Organon theory and practice

**Fig. 1:**
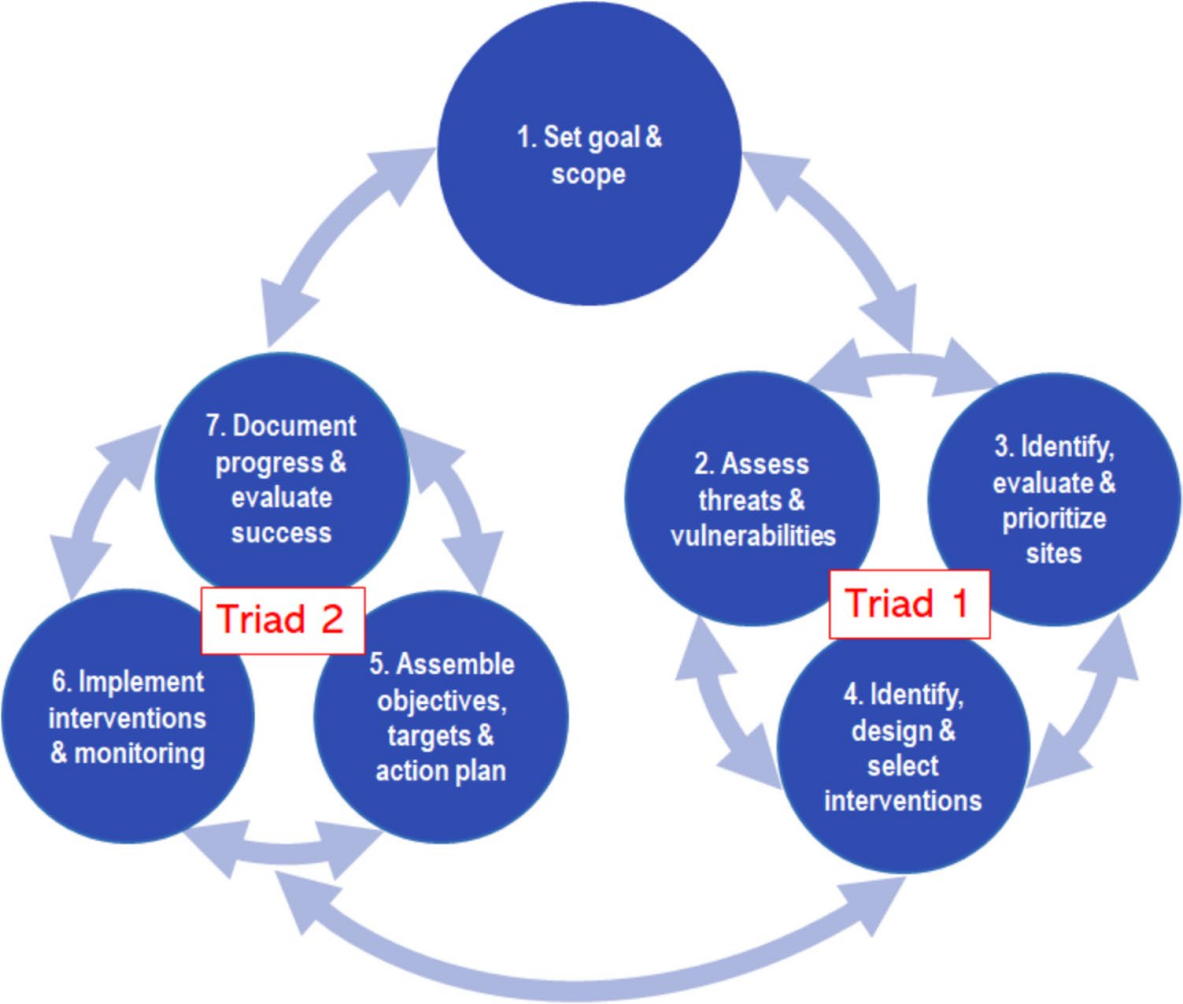
The Organon framework depicts the iterative flow of information between planning science (Triad 1) and implementation science (Triad 2) based on a clearly defined goal and scope (U.S. EPA 2024)

Synthesized from decades of EPA and partner research projects on environmental change impacts and adaptation in aquatic ecosystems, the Organon takes the form of an interactive information system (U.S. EPA 2024) that contains an organizing framework (Fig. 1), guiding principles, project examples, and information resources for integrating resilience and adaptation into environmental management. A foundational principle is that, in the context of a clearly defined goal, achieving resilience requires coordinated flow of information between the science of planning (Triad 1) and the science of implementation (Triad 2) to inform continuous evaluation and improvement of adaptive management outcomes. While the cycle elements are numbered for ease of referencing, the double-sided arrows and spinning triads emphasize the multi-directional, highly fluid and iterative nature of the process, where advances in steps can be pursued in any order to power new refinements in other steps.

The Organon builds on concepts from other frameworks. Allen et al. (2011, p. 1340) differentiated structured decision-making (problem definition with option selections based on trade-offs) and adaptive management (implementation followed by monitoring, evaluation, and adjustment). The adaptive process involves learning by doing through repeated iterations of these activities (Williams and Brown 2018, p. 996). While social learning and stakeholder collaboration are often noted as important for planning and adaptive management (Dreiss et al. 2017, pp. 218–229; Pahl-Wostl 2007, p. 56; Lynch et al. 2022, pp. 45–56; Yap et al. 2025, p. 7), how and when to include stakeholders in the process is unclear. Some scholars limit stakeholder engagement to consultation on management options (Garmestani et al. 2023, pp. 800–807) while others identify how stakeholders can be involved in the whole process (Di Fant et al. 2025, p. 10–12).

The Organon expands upon and thus differs from these and other sister approaches such as climate-smart conservation (Stein et al. 2014), the RAD framework (Lynch et al. 2022) and steps to resilience (Gardiner et al. 2022). First, it places greater emphasis on the practical necessity of ongoing, internal iterative feedbacks within and between elements of planning science and implementation science (see the spinning triads of Fig. 1) as research and decision-making evolve through time. Second, the seven steps of the Organon are paired with specifically tailored principles for engaging in inclusive collaborations of scientific experts with community partners, whereby iterative feedback loops at each step determine where and when to incorporate and document the effects of community feedback. Thus, the structured Organon process provides a way to track project effects on science and community capacity building. These characteristics help improve our ability to address the combined challenges noted by Susskind et al. (2012, p. 48) that cause failures in collaborative adaptive management by ensuring: clearly articulated goals and objectives, explicit protocols to promote shared learning, use of practical tools and incentives to foster participation, and procedures for managing programs adaptively and cultivating long-term capacity building.

### Uses and content of the Organon

**Fig. 2:**
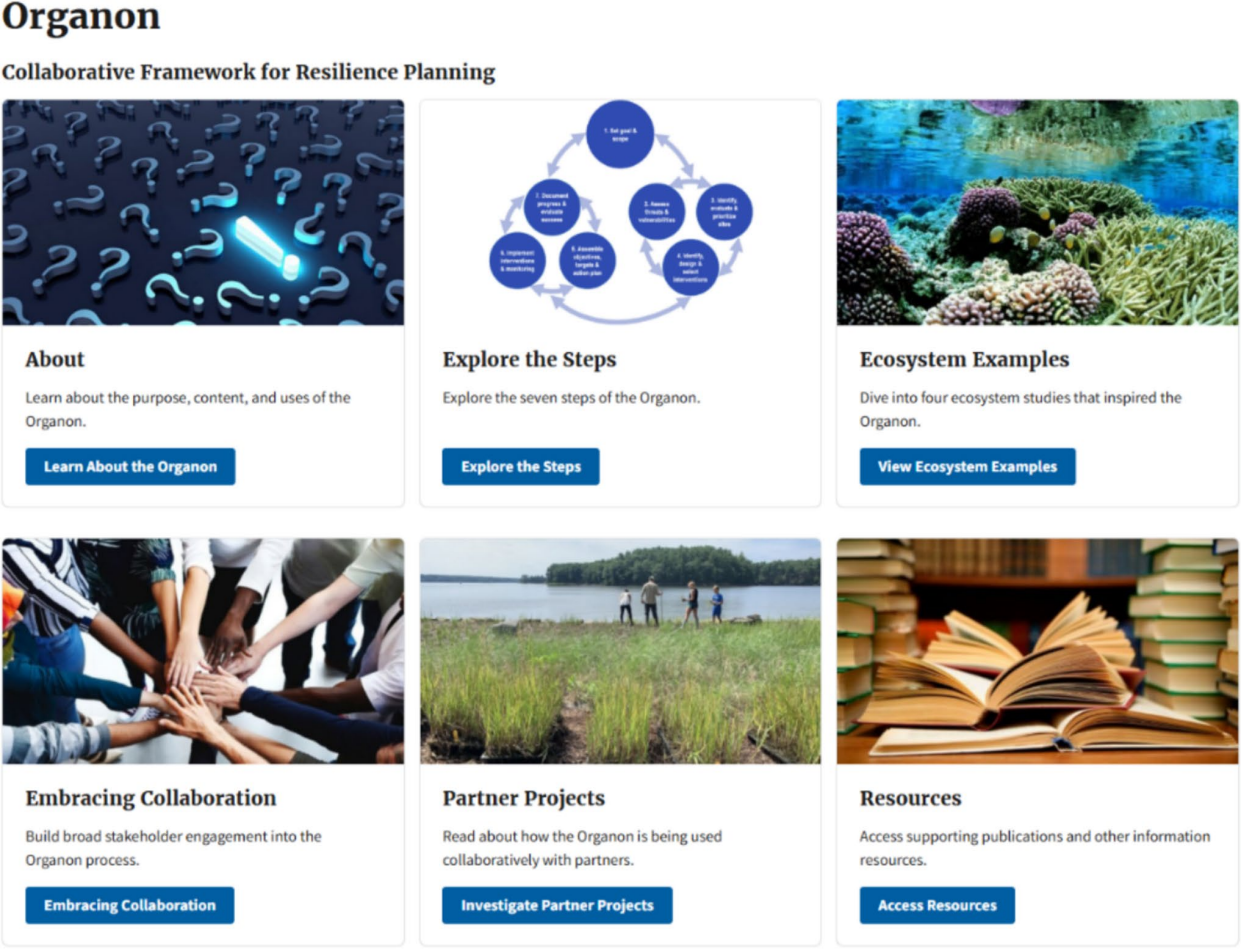
Home page of the Organon website (U.S. EPA 2024)

The EPA Organon has a variety of uses, as illustrated by the content areas of the recently-published Organon website (Fig. 2; U.S. EPA 2024). At the level of general education, the Organon can be explored by anyone interested in an introduction to the key components and basic processes of adaptive planning and action. Users can “Explore the Steps,” by clicking on any bubble in the Organon framework (Fig. 1) to see a general explanation of the terms, underlying principles, and relationship of each step to other steps. For deeper study, users can delve into “Ecosystem Examples” that summarize four previous EPA projects that informed the creation of the Organon and provided the grounding for its unified structure and process. Each ecosystem example (coral reef restoration, cold water fish habitat, stream monitoring networks, salt marsh restoration) contains a narrative description along with links to publications and other resources for those interested in how each study embodied each Organon step.

An equally important counterpart to the environmental sciences in the Organon is incorporation of the social sciences for “Embracing Collaboration” (Fig. 2), i.e., continuous community engagement and public participation. Partnering with local leaders, interest groups, community members, and experts supplements scientific efforts with place-based knowledge, ensures informed decision-making as the work evolves, and fosters community ownership of results. Within this area, users can learn about how to build broad partner engagement into projects through resources covering an introduction to collaboration and step-by-step Organon collaboration best practices.

The combined application of the above is intended to aid more effective program evaluation and project planning. Managers can use the Organon as a programmatic organizing framework to help brainstorm improved adaptation of existing work, review what work is already occurring or still needed, and examine how internal work links together across steps. Project leaders can use it as a logic model with subject matter experts to guide a detailed design process across all steps to achieve a specific project goal. These types of uses are being explored through the “Partner Projects” summarized as follows.

### Partner projects: the Organon in action

**Fig. 3:**
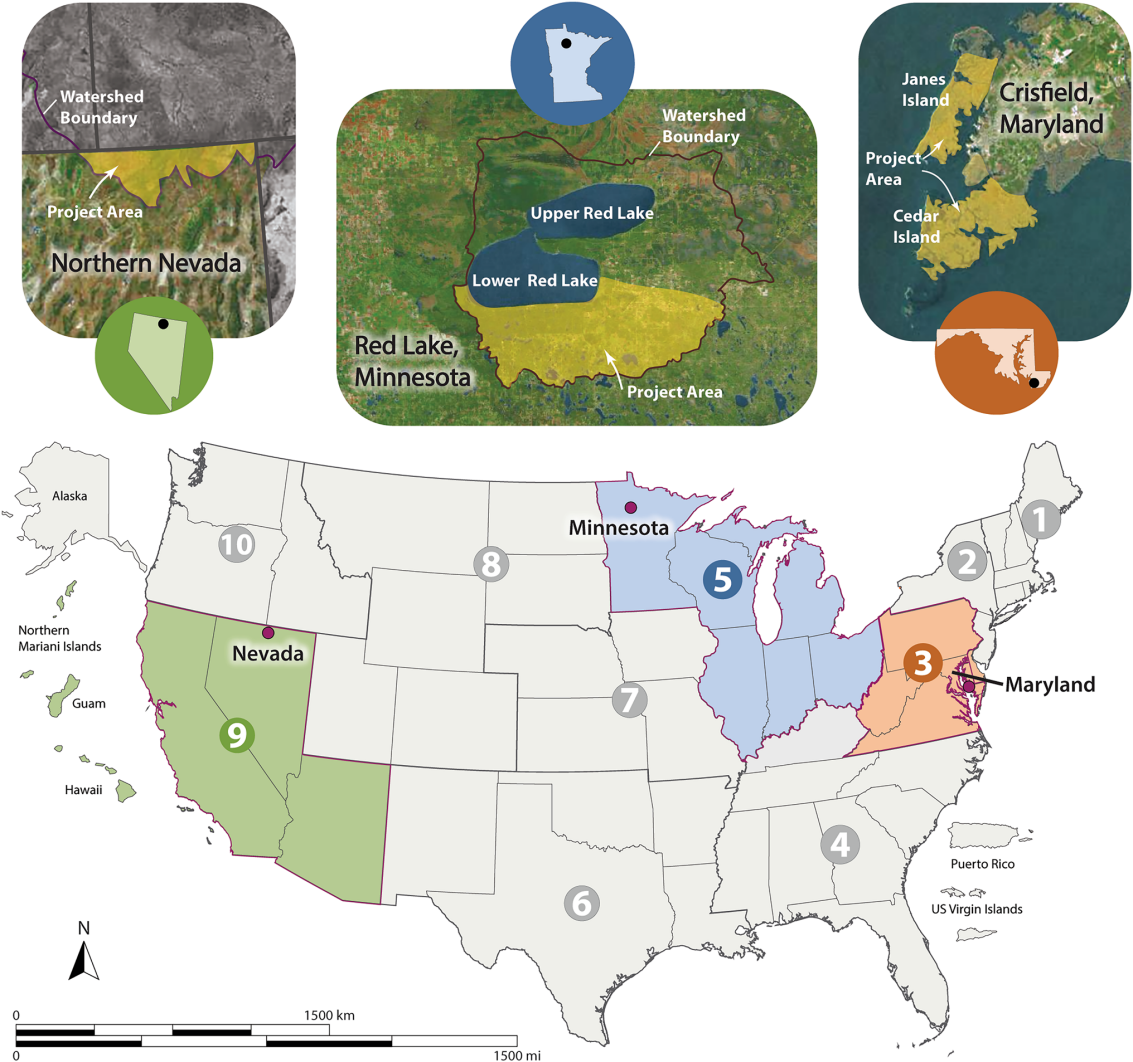
U.S. map showing Organon partner projects. States are grouped into ten EPA Regions (placement of Alaska and Pacific/Caribbean Islands not to scale). Projects are located in Nevada (Region 9), Minnesota (Region 5), and Maryland (Region 3) (see blowout maps identified by state symbols). The Nevada project is just one example from the Region 9 Wetland Program Development Grants

The Organon is being applied in collaborative partnerships across the USA for program and project planning (Fig. 3). Ongoing projects in the “Partner Projects” area of the website include efforts at different scales and levels of decision-making that are using the Organon structure and approach in diverse ways. These projects highlight how the Organon is being applied to support EPA Regions and their state, Tribal, and local government partners in their resilience planning efforts. A brief listing of the projects follows, with additional details about approaches and outputs provided in Table 1.

- Grant writing recommendations: In support of EPA Region 9 states and Tribes, the Organon is being used to provide ideas for writing stronger applications for Wetland Program Development Grants (U.S. EPA 2023) with respect to resilience and collaborative partnership priorities. Focusing on planning steps 1–5 as most relevant to Wetland Program Development Grants, examples for each Organon step illustrate ways that applicants can strengthen their program plans for greater resilience and stronger collaborations.
- Framing adaptive Tribal stream monitoring and management: In collaboration with Tribal scientists of the Red Lake Nation of Minnesota (Red Lake Nation 2025) in EPA Region 5, the Organon is being used to co-develop an adaptive strategic planning framework for stream monitoring and management. The goal is to develop a more holistic picture of how different pieces of Red Lake’s programs fit together within and across Organon elements and identify areas where their water resource programs can work together more efficiently and better use monitoring data for most effective management.
- Flow modeling for iterative solutions-driven research: In support of a resilience-building project with the city of Crisfield, Maryland (City of Crisfield 2023) in EPA Region 3, the Organon is functioning as a flow model to organize and integrate diverse multi-disciplinary research components to assess nature-based solutions for community decision-making. The goal is to help this coastal community increase their resilience to environmental hazards through tailored information on trade-offs of different nature-based solutions for mitigating the impacts of waves and storms while also providing other benefits such as fishing and tourism.

**Table 1:** Partner projects using the Organon are co-developing technical information and transferable tools for resilience-based management of valued aquatic resources

	Grant writing recommendations	Framing adaptive tribal stream monitoring and management	Flow modeling for iterative solutions-driven research
Location	Nevada (Fig. 3 example), California, Arizona, and Pacific Island States/Territories (EPA Region 9)	Red Lake, Minnesota (EPA Region 5)	Crisfield, Maryland (EPA Region 3)
Collaboration goal	Update the guidance for Wetland Program Development Grants to aid applicants with integrating ecological resilience and community engagement in their proposals	Develop a strategic evaluation framework with Tribal scientists of the Red Lake Nation for adaptive stream monitoring and management	Organize and integrate diverse research components to inform community decision-making on nature-based solutions (NBS) for coastal resilience
Process	Use the Organon’s planning components to: ∙ Categorize information in EPA Office of Water’s Core Elements Framework in the areas of: 1) monitoring and assessment; 2) regulatory approaches; 3) voluntary restoration and protection; and 4) water quality standards for wetlands ∙ Connect core elements to specifications for resilience planning and broad public engagement and provide as guidance in the Request for Applications instructions	Use the Organon to holistically frame how: ∙ Program elements such as staffing, funding, and reporting cover the Organon steps ∙ Monitoring data are being/can be used to inform management activities ∙ Individual steps and flows among steps can be improved for more efficient and effective management outcomes ∙ The Tribe can build on strengths and identify needs/next steps for future work	Use the Organon as a flow model to: ∙ Identify how various research components can inform a combined assessment of NBS options for storm surge attenuation and other benefits ∙ Iteratively assess status, progress, and linkages among research components and develop timelines for completion ∙ Develop strategic design considerations to support the community in evaluating NBS options to implement
Outputs to partners	∙ Guidance on key components of resilience-based planning in the context of Wetland Program Development Grants for Core Elements options ∙ Table in the Request for Applications featuring specific examples of how to address resilience and broad public engagement to strengthen applications	∙ Strategic evaluation framework and program analysis for adaptive stream monitoring and management ∙ Collaboration plan to ensure broad participation and promote open communication and transparency ∙ Project-tracker spreadsheet to document project successes, challenges, etc	∙ Analyses of which NBS options have the greatest potential to serve the resilience needs of the community ∙ Visualizations of trade-offs in terms of effectiveness for coastal resilience, co-benefits, feasibility, etc ∙ Strategic design considerations for long-term effectiveness and resilience
Transferable tools	“Fillable table resource” that can be downloaded, edited and adapted for use in other grant application processes	“Strategic evaluation framework” that can be downloaded to serve as a template and informational starting point for other users	“Evaluation table” that can be downloaded and adapted by other users for comparing NBS trade-offs

See Fig. 3 for maps of project locations

## Future directions for using the Organon

The Organon continues to grow and evolve as it is tested by website users and refined based on projects with early adopters. The above examples demonstrate application of the Organon in the context of projects rooted in ecology and environmental change adaptation to advance the EPA’s mission and the needs of EPA regions and their constituents. We have achieved a considerable amount thus far in our current projects by co-developing practicable outputs such as technical guidance, program analyses, strategic designs, and transferable tools that provide benefits to not only the original partners but to other users as well. Yet insights learned to date are also inspiring ideas for new areas, revealing the potential for a broader utility within the EPA as well as externally as a source of guidance, tools and case studies for other organizations. Specifically, we are expanding Organon usage into the areas of public health and developmental evaluation.

### Strengthening public health with the Organon

Adaptation and resilience are fundamental components of protecting human health and the environment (Ebi 2016, p. 3), both within the context of the EPA’s mission and internationally (Hess et al. 2012, pp. 171–172). Hence, there is rich potential to incorporate this overall area within Organon work, emphasizing the relationship of environmental health concerns such as infectious disease, water pollution and waterborne pathogens, health effects from wildfire smoke exposures, and mental health impacts. The Organon’s structured iterative process lends itself well to the ever-evolving characteristics of public health as a practical tool for understanding and explaining the interdisciplinary nature of these relationships, dynamics, and connections to future work.

As with the natural resource examples above, the Organon could be useful for organizing public health concepts associated with ecological models and within adaptation planning. It can be applied toward identifying key stakeholders, determining metrics of success, and other areas of measurement. The following thought experiment applies the environmental health concept of *Total Worker Health*^*®*^ (*TWH*^*®*^*;* a framing of holistic approaches bolstering all aspects of worker well-being; U.S. CDC 2024). We use this to examine how watermen in the Chesapeake Bay increasingly may be affected by marine pathogens due to environmental changes, and how to incorporate watermen’s mental health in relation to an adaptation strategy for resilience.

For context, research has shown that the *TWH®* of watermen, like other agricultural workers, has ancillary relationships with access to healthcare, including mental healthcare; occupational health and safety; food and economic security; and other environmental health stressors (Gould et al. 2024, pp. e637–e640). The physical and socioeconomic health of these workers may be impacted by waterborne pathogens such as *Vibrio parahaemolyticus* and *V. vulnificus*. Increased prevalence of vibriosis disease is occurring as bacterial propagation and extent are driven by warming sea surface temperatures and longer warm seasons (Sheahan et al. 2022, pp. 087007-5–087007-7). Disease occurs following ingestion, usually of raw or undercooked shellfish, or dermal exposures from open wounds coming into contact with contaminated water. Physical health outcomes from vibriosis include “food poisoning” symptoms as well as more serious effects like necrotizing fasciitis, sepsis, and death (Baker-Austin et al. 2018, p.1). Beyond physical impacts, watermen’s *TWH*^*®*^ may be impacted by lost income resulting from an inability to work or economic effects from consumer illness or death, and anxiety about causing harm to others or an inability to provide for one’s family, all of which may have short- or long-term effects on mental health (Gould et al. 2024, p. e638). While this is a U.S.-focused example, it could be extrapolated to other parts of the world given the prevalence of *Vibrio* internationally (Le Roux et al. 2015) and difficulties for any fisheries workers in avoiding waterborne or consumer-driven diseases (Change-Chien et al. 2007, p. 616; Freitag et al. 2022, p. 562–564; Gould et al. 2024, pp. e637–e640).

Table 2 applies an Organon approach to a hypothetical project to improve the *TWH®* of watermen in the Chesapeake Bay region in the context of environmental change-exacerbated *Vibrio-*related threats and adaptation planning. (For a more detailed table that also includes Organon step definitions, see Online Resource 1/Table 3) We start with a general goal that, after an initial iteration of steps 2–4, is refined to a Specific, Measurable, Achievable, Relevant, Time-bound (SMART) goal (Doran 1981, p. 36): *By 2030, improve the TWH*^*®*^* of Chesapeake Bay watermen based in Maryland and Virginia with respect to vibriosis, as measured by their perceptions of work-related physical and mental health strain and personal and commercial stability and security.*

**Table 2:** Organon components (steps) of planning and implementation illustrated with a hypothetical public health project on *Total Worker Health*^*®*^* (TWH*^*®*^*)* of watermen impacted by *Vibrio* spp.-related illnesses in the U.S. Chesapeake Bay region (adapted from U.S. EPA 2024 )

Organon steps	Chesapeake bay watermen example
1. Set goal & scope	Initial (general) goal and scope: Reduce the negative impacts of *Vibrio* spp. on the health and well-being of watermen of the Chesapeake Bay region Second-iteration (SMART*) goal and scope after first-round planning (steps 2–4): By 2030, improve the *TWH*^*®*^ of Chesapeake Bay watermen based in Maryland and Virginia with respect to vibriosis, as measured by their perceptions of work-related physical and mental health strain and personal and commercial stability and security *Specific, Measurable, Achievable, Relevant, Time-bound
2. Assess threats & vulnerabilities	Threats to the watermen community: Vibriosis through dermal exposures (wound infections) or ingestion (food borne illnesses) Vulnerability depends on: ∙ Exposure—risk of vibriosis exposure increases under increasingly warmer water conditions ∙ Sensitivity—sensitivity is affected by pre-existing health conditions, lack of effective personal protective equipment (PPE), inconsistent wound treatment, limited access to healthcare and changes in consumer behaviors ∙ Adaptive capacity—can be increased via training programs to increase skill sets for protection and programs to refine/expand interventions Vulnerability can be decreased by reducing exposure, reducing sensitivity, and/or increasing adaptive capacity Data gaps: limited data on where/how exposures occur; need improved forecasting of bacterial levels, including environmental change effects
3. Identify, evaluate & prioritize sites	Sites can be viewed as the different boats on which the watermen work and the different areas where they fish, and locations of consumer exposures Evaluation of the vulnerabilities at these “site” levels (e.g., what fishing areas have the most *Vibrio* and which boats could benefit from improved safety measures) will have implications for the interventions (step 4), and how to design them Sites could be prioritized based on their different suitabilities for implementing activities Data gaps: need improved spatial monitoring/predictive capabilities of bacteria in water and shellfish, information on safety protocols on boats, and treatment protocols post-infection
4. Identify, design & select interventions	Potential interventions to date have included, for example: ∙ Laminated information sheets (including information on proper use of PPE) for shipboard posting ∙ A restaurant and consumer awareness campaign on safe shellfish storage and early detection of vibriosis symptoms ∙ Awareness campaigns to destigmatize prioritization of mental health among watermen and increase awareness among healthcare providers As understanding of site-specific vulnerabilities increases, consider design of interventions (existing and novel) to increase effectiveness This could affect selection of interventions to focus on first, in a particular order, or in certain places, for biggest “bang for the buck” Data gaps: need info on how and where interventions are being used, and how their designs could be improved/adapted to changing conditions
5. Assemble objectives, targets & action plan	Objectives accompanied by measurable targets for reaching those objectives could include, for example: ∙ Within a specified timeframe, develop laminated information sheets to post on boats and in shellfish processing facilities on how to limit exposures, clean wounds, use PPE, recognize vibriosis symptoms, and seek medical treatment ∙ Work with sanitation organizations and hospitality associations to create an advertising campaign on food safety measures for eating shellfish, sharing risk factors and symptoms of vibriosis and personal and community-wide impacts ∙ In conjunction with medical associations and departments of public health, develop an outreach and education effort targeting healthcare professionals that emphasizes checking patient mental health during exams, especially those who work on the water An action plan consisting of practical elements of how—and by whom—the work would be accomplished would: ∙ Identify and engage key actors to provide recommendations on language, timelines, budgets, and other necessary resources
6. Implement interventions & monitoring	The project would implement interventions through focused pilot projects, with monitoring throughout the project to measure effectiveness at the levels of both process (were actions implemented as planned) and outcomes (were risks reduced as targeted) For this theoretical project, this would mean an initial focus on a smaller subset of the effort as laid out in the action plan, such as: ∙ A certain subsample of watermen operations across the region ∙ One part of the region (e.g., watermen based in Somerset County, MD) Based on pilot results, adjustments could be made to the action plan (including going back to Triad 1 for more info), followed by full scaling-up
7. Document progress & evaluate success	Documenting progress on practical implementation of intervention activities could include, for example: ∙ Interviewing watermen and healthcare providers on perceptions of change in risk of adverse health outcomes, including mental health needs ∙ Conducting annual statistical analyses to determine zip codes and scenarios in which exposure occurred and detect patterns Evaluating success in terms of the ultimate desired outcomes would involve measuring, for example: ∙ Changes over time in how watermen perceive their livelihoods and income being affected by vibriosis cases and resultant effects ∙ The extent to which watermen feel comfortable discussing and seeking treatments for mental health concerns with their healthcare providers ∙ The extent to which healthcare providers report feeling knowledgeable regarding occupational health threats to watermen, writ large ∙ Changes in the number of vibriosis cases from consumption of raw or undercooked seafood Based on feedback and results, consider how implementation steps best meet the needs and expectations of stakeholders (e.g., watermen, public health officials, healthcare professionals) and revise steps as necessary to improve them

Iteration: Note that the entire process is iterative, where advances in any one step can power further refinements in other steps; for example, the evaluation process–particularly during pilot projects–may reveal weaknesses or opportunities to change previous elements to better meet stated goals and objectives

Table 2 outlines how the project would identify threats posed by *Vibrio* spp. to the watermen, including how *Vibro* spp. prevalence is driven by large-scale environmental changes, with assessment of vulnerability based on the degree of exposure to the threats, plus the sensitivity and adaptive capacity of the watermen and their social systems for resistance or recovery. Based on this information, the project would focus on specific sites and situations in which exposures to *Vibrio* spp. occur, ascertain resources and gaps, and make recommendations for interventions. A first pass through these “Planning Triad” steps—to review existing knowledge and identify priority needs—is what allows for refining the original general goal into a SMART goal, the specificity of which will drive more detailed planning iterations and a more powerfully-focused action plan. The action plan would lay out the specific objectives and targets for piloting and then scaling-up the selected interventions that are designed to improve the *TWH*^*®*^ of watermen in the context of environmental change-exacerbated vibriosis threats and evaluating whether the SMART goal has been achieved.

While this example is specific to one occupational population within the U.S. Chesapeake region, the same approach could be extrapolated to other public health topics, industries, or parts of the world. The flexibility of the Organon framework allows for retaining information that may be the same, swapping out information that is different, and benefitting from lessons learned from using a consistent framework of steps and principles.

### Evaluating socio-ecological practice research with the Organon

Transparency and detailed documentation of complex, collaborative planning and implementation processes promoted by the Organon lay the basis for developmental evaluation. The “Embracing Collaboration” component of the Organon is a central mechanism that drives the iterative planning and implementation steps. Interdisciplinary collaboration improves the science needed to solve complex socio-ecological challenges, and collaborating with stakeholders and local communities improves scientific efforts through local knowledge, community ownership, and capacity building for improved project sustainability. Evaluation techniques to assess co-created research projects are still new (Brix et al. 2020, p. 170), and while community capacity building is an understood goal of such research, work assessing capacity building outcomes from collaborative research is limited.

Interdisciplinary collaborative research in complex socio-ecological systems requires an evaluation approach that can adequately consider context, process, and change. Traditional evaluation approaches include summative evaluation, which occurs at the end of a project to assess merit or worth of efforts against initial goals and objectives, and formative evaluation, which involves an ongoing process of assessing performance for goal alignment (Bamzai-Dodson and McPherson 2022, p. 3; Gamble 2008, p. 15). While useful, these approaches do not allow for continuous change and ongoing development of research goals and objectives through collaboration and co-learning with project partners. *Developmental* evaluation tracks changes in goals, objectives, and project focus in response to relationship building and co-learning as the interdisciplinary team communicates with each other and with stakeholder partners to ensure cohesive, locally relevant, and usable science (Gamble 2008, p. 15; Patton 2011, p. 11). In this way, developmental evaluation captures the “Embracing Collaboration” component of the Organon while the Organon adds a structured framework to guide what to document and when, and how to link processes through analysis to explain outcomes.

**Fig. 4:**
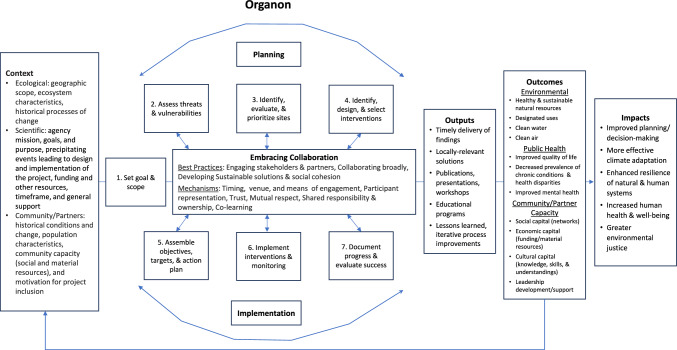
A proposed model to evaluate co-production and community capacity building, adapted from Brix et al. (2020, p. 175)

The diagram in Fig. 4 was adapted from a generic program theory for co-production (Brix et al. 2020, p. 175) to accommodate the feedback effects among the seven steps and “Embracing Collaboration” best practices of the Organon. Each box includes a non-exhaustive list of concepts to consider. The model begins with context. Context sets a basis for understanding changes in project goals, objectives, and processes and supports comparison with other projects to explain variations in outcomes. Contextual factors span levels and scales of ecological goals, community/stakeholder partners, and science/project team characteristics. The ecological context informs the environmental issues in need of a solution and sets the parameters for identifying the geographic and social boundaries of the project and science support needs. For example, the ecological context of the watermen example noted above is warming sea surface temperatures and longer warm seasons, which are leading to increases in waterborne pathogens such as *Vibrio parahaemolyticus* or *V. vulnificus* and *Vibrio*-related illnesses in the Chesapeake Bay region (Muhling et al. 2017, p. 278).

The scientific context involves employer mission, goals, and purpose, employee roles and responsibilities, precipitating events leading to design and implementation of the project, team dynamics, funding and other resources, timeframe, general support (including human capital), and the sciences represented. As in the case of the watermen example of Gould et al. (2024), which was completed in a university context, grant funding, tenure status of faculty, faculty time allocation for the project, and graduate student or other research assistant support would be important considerations in addition to scientific expertise relevant to watermen health, such as public health, medicine, and molecular pathobiology. Additional partnerships with Sea Grant and/or state fisheries agency scientists would add a connection to local circumstances and pathways for interventions.

Community/partner context involves historical conditions and change, population characteristics, capacity (social and material resources), and motivation for project inclusion. In the watermen case, the geographic context and specific shellfish fisheries affected by *Vibrio* spp. indicate who is at risk of vibriosis disease and the pressures that increase risks, as well as resources useful for intervention pathways. Resources include social capital (e.g., status, funding, and skills accessible through social relations), economic capital (e.g., money, credit, and technology), and cultural capital (e.g., skills, ways of knowing, and motivation) (May 2022, p. 45). Complexities arise due to pressures from seafood markets, environmental conditions, technological developments, variability in resources across sectors, occupation, firm size and operation costs, demographics of fisheries workers, their families, and communities, cooperation, competition, conflict among watermen and sectors, and representation in associations and agency decision-making. These influence the gap between resources available and needed, motivations, capacities, and cost–benefit analyses regarding willingness to accept risk and implement risk reduction strategies (May 2012, p. 414). Hence, context is important for identifying the people affected by and interested in the issue, recruiting community partners and other stakeholders, and ensuring a proper fit between the issue addressed and scientific expertise represented. In this sense, context directly shapes the initial goal and scope of the project, which are further refined through iterative feedback effects between the “Embracing Collaboration” mechanisms and each step of the planning and implementation cycles.

The factors listed as mechanisms in the “Embracing Collaboration” box set the process of collaboration in motion. These mechanisms include timing, venue, and means of engagement and representation in project development, shared responsibility, participation, trust, reciprocity, respect, shared ownership, and learning. For example, watermen live and work according to the tides and weather, have historically distrusted scientists and government, and represent diverse interests often in competition for resources and recognition (May 2012, p. 409). Sensitivity in planning the when, where, and who of engagement practices can improve the comfort level of participants, perceptions of respect, and development of trust over time through iterative feedback processes and relationship building among project team and stakeholder partners. Trust and respect across representative groups increase the effectiveness of project design and implementation, particularly where interventions require behavioral modifications with material and/or non-material costs.

Theoretically, relationship building that involves development of trust and respect in knowledge exchange and co-learning leads to emergent processes of community capacity building, as per Emery and Flora’s (2006, p. 22) “spiraling-up” of capitals. “Spiraling-down” (contraction of capital resources) often follows socio-economic, demographic, technological, natural, or other shocks and disruptions, with cascading consequences for community, public, and environmental health. For example, watermen in the Chesapeake Bay area share similarities with commercial fishers and their communities across the U.S. Market competition, declining fish stocks, fisheries regulations, competition with alternate fisheries uses, technological change, and natural and technological disasters have resulted in the consolidation of fishing firms, i.e., fewer but larger fishing boats (May 2012, p. 409). The consequences are decreasing numbers of fish houses and processors, conversion of commercial infrastructure to recreational uses, and aging of the fishing fleet as the younger generation finds employment outside the industry. As people leave the industry and move away from fishing communities, formal support services for watermen decline, and informal supports from social networks also contract. These conditions limit the ability of remaining watermen to withstand or respond to additional shocks, such as increasing instances of vibriosis disease.

Inclusive planning and implementation processes can initiate a reversal in this type of downward spiral of capital depletion, i.e., a “spiraling-up” of capitals (May 2022, p. 45). Changes in relationships and experiences potentially cause a change in peoples’ social capital, which increases economic capital and skills, knowledge, ways of knowing, and motivation needed for action. For example, inclusive collaboration of scientists with watermen, representatives from fisheries agencies, public health and nongovernmental organizations, and fisheries associations has the potential to broaden relations, increase trust and respect, and thus increase the resources available to reduce the risks of vibriosis. Thus, the trust, reciprocity, and mutual respect fostered by relationship building cause increases, decreases, and qualitative changes to the resources people can access for improved planning and decision-making for enhanced resilience of natural and human systems and improved human health and well-being. However, while collaborative research is assumed to result in improved capacity outcomes for partners and/or communities, little work systematically evaluates these connections (Brix et al. 2020, p. 170).

The developmental model described here supports evaluation of the steps and capacity outcomes of the Organon in relation to context and collaboration. Documentation of each step and communication among project team members and stakeholders promotes transparency and allows for analysis of the iterative processes and feedback effects that shape project results across distinct time periods. The non-exhaustive concepts noted in the model offer structured yet flexible factors that could be measured and the temporal order for tracking and assessing change and effects on project processes and outcomes. Current EPA efforts to evaluate co-production and capacity building for coastal community resilience in the Chesapeake Bay include analyses of email correspondence, meeting minutes, reports, and interviews with science team and community partners. Documenting progress and change across the lifetime of a project allows for reflexivity and intentionality in corrective measures for goal realignment, resource use and needs, and course corrections as new knowledge is incorporated. A major benefit of the detailed documentation process is a more complete understanding of lessons learned that can inform projects across issue domains and contexts. Furthermore, the developmental model proposed here is sensitive to how outcomes are likely to change contexts and create opportunities to adaptively manage evolving circumstances and emerging issues over the course of project planning and implementation.

## Conclusions: advancing holistic resilience planning using the Organon

The Organon and its application in a variety of partner projects is advancing the science of holistic resilience-based adaptation at the EPA. It demonstrates a set of essential attributes and mechanisms necessary for addressing complex issues in the context of the highly integrative, fluid, and ever-changing nature of scientific assessments and social decision-making. Specifically, the Organon provides:

- A *structured process* that explicitly recognizes the major steps in operationalizing scientific and technical knowledge in adaptation planning and implementation contexts and allows for those steps to be systematically applied.
- *Best practices* and* transparency* within and across each element of the structured process, to bolster collaborative relationships, create accountability, and enable formal evaluation of processes and outcomes.
- Built-in *iterations* (as a function of complexity of project, number and type of partners, scale, etc.) to leverage the structure and transparency to create internal learning and an improved match between scientific capabilities and stakeholder needs.

For the broader community of practice, an “organon” could take many other forms. The EPA Organon is tailored to improve forward-looking, robust adaptation science and decision-making in the context of the EPA’s mission to protect human health and the environment. Its terminology and steps are intended to be flexible and adaptable for use in a variety of sectors and systems within the EPA’s purview. Other organizations might design their own version of an organon, but we assert that every organization working in the adaptation space should have some kind of organon. Adaptation work is messy and meandering due to constantly changing information and stakeholder needs. Many projects continue to be laid out as a tidy sequence of steps on a linear timeline, when in reality they transpire in a nonlinear way, with operational loops and iterative refinements of both the science and stakeholder interactions and decision-making occurring repeatedly. This often plays out invisibly in a “black box,” without the documentation needed to allow process evaluation to improve effectiveness and efficiencies. An organon approach is aimed at improving our collective ability to more rigorously guide, inform, coordinate, implement, document, and evaluate progress, as well as learn from collaborative work with partners to achieve more effective resilience-based adaptation for people and the environment.

## Supplementary Information

Below is the link to the electronic supplementary material.

Supplementary file1 (DOCX 62 KB)

## Data Availability

No datasets were generated or analysed during the current study.

## Footnotes

1 Originating as a term for Aristotle’s collective works on logic, “organon” is now used to refer to any instrument or tool for acquiring knowledge or conducting scientific and philosophical inquiry. More recently it is in use by a company called Organon that links the meaning to an instrument for advancing women’s health.
