# Supplementary material for: Planning and managing for resilient natural resources and communities in the USA: the EPA Organon
Source: Socioecol Pract Res. 2025 Aug 7;7(3):275–88. doi: 10.1007/s42532-025-00224-1 (PMC12589289; doi:10.1007/s42532-025-00224-1)
Supplement: Supplementary file 1 — Supplementary file1 (DOCX 62 KB) [file 42532_2025_224_MOESM1_ESM.docx]

**Online Resource 1**

**Table 3** Organon components (steps) of planning and implementation, with full step definitions illustrated by a hypothetical public health project on Total *Worker Health® (TWH®)* of watermen impacted by Vibrio spp-related illnesses in the U.S. Chesapeake Bay region (adapted from U.S. EPA 2024)

| **Organon Steps** | **Description** | **Chesapeake Bay Watermen Example** |
| --- | --- | --- |
| **1.**  **Set Goal & Scope** | A clearly articulated **goal** is one that specifies the desired effect of a management effort in terms of what is to be achieved ecologically and socially, and over what time frame (years to decades)  The **scope** of the effort refers to the extent of area (geographic boundaries) and/or framing of social parameters (such as human economic brackets or community types) on which the work will focus | **General**  Reduce the negative impacts of *Vibrio* spp. on the health and well-being of watermen of the Chesapeake Bay region  **Second-iteration (SMART*) form after first-round planning (steps 2-4)**  By 2030, improve the *TWH*® of Chesapeake Bay watermen based in Maryland and Virginia with respect to vibriosis, as measured by their perceptions of work-related physical and mental health strain and personal and commercial stability and security  ***S**pecific, **M**easurable, **A**chievable, **R**elevant, **T**ime-bound |
| **2.**  **Assess Threats & Vulnerabilities** | An early critical task is to **assess threats** to the natural resource and associated human communities from local stressors, both natural and anthropogenic, and their interactions with large-scale environmental change stressors  The next stage is to **assess vulnerabilities** based on the degree of exposure to the threats plus the system's sensitivity and adaptive capacity to resist or recover | **Threats** to the watermen community: Vibriosis through dermal exposures (wounds or open sores) or ingestion (food-borne illnesses)  **Vulnerability** depends on:   - Exposure—risk of vibriosis exposure may increase under increasingly warmer temperatures and longer warm seasons (some watermen already avoid warmer months for harvesting) - Sensitivity—sensitivity is affected by pre-existing health conditions but also by lack of effective PPE, inconsistent wound care treatments and other related protocols, limited access to healthcare including mental healthcare, limited time to seek treatment, lack of medical insurance, and changes in consumer behaviors - Adaptive capacity—adaptive capacity can be increased via training programs to increase skill sets for protection and programs to refine and expand interventions   **Vulnerability** can be decreased by reducing exposure, reducing sensitivity, and/or increasing adaptive capacity  Data gaps: data regarding where and how exposures occur are limited; need improved forecasting of bacterial levels, including expected effects of environmental change |
| **3.**  **Identify, Evaluate & Prioritize Sites** | Within the defined geographic and social scope, it is necessary to **identify site(s)** that are smaller spatial units suitable in scale for implementing management projects or activities; in some cases, a single site may already be the focus  The need to **evaluate site(s)** recognizes that sites can vary in their suitability for different interventions based on criteria such as relative contribution to the goal, current and future status of the resource, vulnerability, and management context  Decision-making to **prioritize site(s)** for piloting and potential scaling-up of management activities to achieve the goal is based on review and discussion with appropriate management, experts, and other stakeholder groups | **Sites** of exposure to *Vibrio* can be viewed as the different boats on which the watermen work, the different areas where they fish, and fishing and harvesting gear. Furthermore, when considering mental health, one can include sites of consumer exposures upon consumption  **Evaluation** of the vulnerabilities at these “site” levels (e.g., *in situ*, real-time *Vibrio* concentrations, spot-testing of raw shellfish in restaurants and markets prior to serving, restaurant and consumer awareness, healthcare worker awareness of vibriosis symptoms, sequelae including implications for human health) will have implications for the interventions (step 4), and how to design them  Sites could be **prioritized** based on their different suitabilities for implementing activities  Data gaps: need more improved spatial monitoring and predictive capabilities of bacteria in the water and in shellfish, improved information on safety protocols on boats and treatment protocols post-infection |
| **4. Identify, Design & Select Interventions** | Proceeding to **identify interventions** involves brainstorming a full list of all available management actions that could be taken to address threats and vulnerabilities at sites selected as key to achieving the management goal  Before making any choices, it is useful to assess how to **design interventions** by adapting components, configurations, timing, and other elements to ensure robustness under changing environmental conditions  This makes it possible to **select interventions** based on evaluation criteria that include design effectiveness under both current and future conditions, along with other key determinants such as cost, feasibility, urgency, flexibility, and multiple benefits | Potential **interventions** to date have included:   - Laminated information sheets (with info such as proper use of gloves as PPE against dermal exposure) for posting shipboard and in shellfish processing facilities - A restaurant and awareness campaign focused on safe shellfish storage and consumption practices and early detection of vibriosis symptoms - Awareness campaigns to destigmatize the prioritization of mental health among watermen and increase awareness among healthcare providers - Trainings on workplace safety that are incorporated into fishing permitting processes (usually optional)   As understanding of site-specific changing vulnerabilities increases, it may be possible to better **design** interventions to increase their effectiveness in light of changing conditions, as well as invent novel interventions that do not currently exist  This could affect **selection** of interventions to focus on certain ones first, or in a particular order, or in certain places, to get the biggest “bang for the buck”  Data gaps: need info on how and where these interventions are being used, and how their designs could be improved/adapted to changing conditions |
| **5.**  **Assemble Objectives, Targets & Action Plan** | The transition from technical planning (steps 2-4) to practical implementation (steps 5-7) starts with the assembly of precise **objectives** that are Specific, Measurable, Achievable, Relevant, and Time-bound (SMART)  Such precision enables the creation of management **targets** that can function as stepping-stones along a path to achieving the objectives, by describing where and how each work element will be done under a defined implementation timeline  The **action plan** then aggregates the full suite of interventions, objectives, targets, and timelines--tailored by site as appropriate--to guide the practical implementation of management activities to achieve the overall goal | **Objectives** accompanied by measurable **targets** for reaching those objectives could include, for example:   - Within a specified timeframe, collaborating with the Interstate Shellfish Sanitation Conference (ISSC) to develop laminated information sheets in English and Spanish to post on boats and in shellfish processing facilities and restaurants on how to limit exposures and clean wounds *in situ*; use PPE to reduce exposures; recognize basic vibriosis symptoms; and know when and how to seek medical treatment - Working with ISSC members and hospitality associations to create an advertising campaign on food safety measures for eating U.S.-grown shellfish and strength of existing food safety measures, sharing risk factors and symptoms associated with severe vibriosis and info on how lack of care and attention can have significant personal and also community-wide, impacts (e.g., effects on watermen and their families due to lost income) - In conjunction with medical associations and departments of public health for Virginia and Maryland, developing an outreach and education effort targeting healthcare professionals that emphasizes the need to check in with patients on their mental health, especially those patients who work on the water   An **action plan** consisting of practical elements of how—and by whom—the work would be accomplished would:   - Identify and engage key actors needed to provide context and recommendations on language that resonates with target populations, a timeline for development and implementation, budgets for different projects, and other necessary resources |
| **6.**  **Implement Interventions & Monitoring** | To **implement interventions**, the sequence and timing of activities in the action plan are now applied in the field, through initial pilot trials (where needed) that are then scaled-up to fully achieve objectives  **Monitoring** to assess the efficacy of interventions in the initial phase of implementation should be expanded into a more comprehensive monitoring plan that specifies metrics and methods, measures of effectiveness, spatial scale and locations, timeframe, and roles and responsibilities for collecting data at the full scale of the longer-term effort | The project would **implement interventions** through focused pilot projects, with **monitoring** throughout the project to measure effectiveness at the levels of both process (were actions implemented as planned) and outcomes (were risks reduced as targeted)  For this theoretical project, this would mean an initial focus on pilot methods and metrics for a smaller subset of the effort as laid out in the action plan, such as:   - A certain subsample of watermen operations across the region - One part of the region (e.g., watermen based in Somerset County, MD)   Based on the pilot results, adjustments could be made to the action plan (including going back to Triad 1/steps 2-4 for more info if needed), followed by full scaling-up of the effort |
| **7.**  **Document Progress & Evaluate Success** | A key to assessing performance is to **document progress** by tracking the implementation team's practical completion of project installation and other intervention activities as specified in the action plan and associated work plan  Analyses to **evaluate success** will involve comparing monitoring results (changes in metrics) to the objectives laid out in the action plan, where success is reflected by ecological changes that demonstrate concrete advancements toward, and eventual achievement of, the ultimate long-term goal | **Documenting progress** on practical implementation of intervention activities could include, for example:   - Administer a survey during a regional watermen’s association meeting to learn how many watermen teams have attended trainings during the permitting process or posted laminated information and to receive feedback on trainings - Interview watermen on their perceptions of change in risk and adverse health outcomes for themselves and informed consumers and access to knowledgeable and compassionate healthcare workers and mental health supports - Interview and survey healthcare providers to gauge their perceptions of their awareness of vibriosis exposures and symptoms and mental health needs of watermen pertaining to occupational stress - Conduct annual statistical analyses of state-based vibriosis cases to determine zip codes and scenarios in which exposures occurred, to detect patterns   **Evaluating success** in terms of the ultimate desired outcomes would involve measuring, for example:   - Changes over time in how watermen discuss interruptions to their livelihoods and annual income as a result of personal or consumer vibriosis cases and resultant effects - The extent to which watermen feel they may speak to and receive understanding from their healthcare providers about mental health concerns, with one metric being increased numbers of watermen who seek psychological or psychiatric treatment in the form of mental health therapy or prescribing practices - The extent to which healthcare providers report feeling knowledgeable regarding occupational health threats to watermen, writ large, including threats presented by microbial hazards and downstream effects like consumer illness - Changes over time in the number of vibriosis cases and deaths that result from restaurant or market patrons (or similar) consuming raw or undercooked seafood   Based on feedback or results from these steps, consider how the proposed implementation steps best meet the needs and expectations of stakeholders (e.g., watermen, public health officials, healthcare professionals), then revise steps as necessary to improve them |
| **Iteration:**  Note that the entire process is iterative, where advances in any one step can power further refinements in other steps; for example, the evaluation process–particularly during pilot projects–may reveal weaknesses or opportunities to change previous elements to better meet stated goals and objectives | | |
